# Supplementary material for: Analysis of PPARγ Signaling Activity in Psoriasis
Source: Int J Mol Sci. 2021 Aug 10;22(16):8603. doi: 10.3390/ijms22168603 (PMC8395241; doi:10.3390/ijms22168603)
Supplement: Supplementary file 1 [file ijms-22-08603-s001.zip › Supplemental materials_Analysis of PPARg signaling activity in psoriasis/Pathway models/Models images and html files/PPARG inhibits phenotypes assotiated with psoriasis/124162.html]

asthma


# Disease asthma

|  |  |
| --- | --- |
| URN | urn:agi-meshdis:Asthma |
| Total Entities | 15 |
| Connectivity | 8619 |
| Name | asthma |

---

|  |  |
| --- | --- |
| ChildConcepts | cardiac asthma |
|  | aspirin-exacerbated respiratory disease |
|  | allergic asthma |
|  | extrinsic asthma |
|  | intrinsic asthma |
|  | mild intermittent asthma |
|  | mild persistent asthma |
|  | moderate persistent asthma |
|  | nocturnal asthma |
|  | occupational asthma |
|  | severe persistent asthma |
|  | cough variant asthma |
|  | exercise-induced asthma |
|  | status asthmaticus |
|  | experimental asthma |

---

|  |  |
| --- | --- |
| Pathway | ADAM33 Role in Asthma |
|  | Genes with Mutations Associated with Asthma |
|  | Proteins with Altered Expression in Asthma |
|  | PPARG inhibits phenotypes assotiated with psoriasis |
|  | Neighbors of asthma |
|  | Overview. Diseases of the respiratory (chapter 9) |

---

|  |  |
| --- | --- |
| MedScan ID | 9010090 |

---

|  |  |
| --- | --- |
| Alias | late asthmatic reaction |
|  | immediate asthmatic reactions |
|  | chronic asthma |
|  | early asthmatic reactions |
|  | Asthma exacerbations |
|  | late asthmatic response |
|  | Asthmatic responses |
|  | Asthmas |
|  | late asthmatic responses |
|  | early asthmatic reaction |
|  | chronic asthmas |
|  | lung allergies |
|  | asthmatic subjects |
|  | Bronchial Asthmas |
|  | Bronchial Asthma |
|  | exacerbations of asthma |
|  | Asthmatic response |
|  | lung allergy |
|  | immediate asthmatic reaction |
|  | Asthma |
|  | asthmatic subject |
|  | asthma pulmonale |
|  | asthmatics |
|  | Asthma exacerbation |
|  | atopic Bronchial Asthma |
|  | childhood asthma |
|  | late asthmatic reactions |
|  | early asthmatic response |
|  | exacerbation of asthma |
|  | Asthmatic |
|  | early asthmatic responses |
|  | Asthma Unspecified |
|  | immediate asthmatic responses |
|  | Asthma symptoms |
|  | asthma bronchiale |
|  | Asthma, Bronchial |
|  | symptoms of asthma |
|  | Bronchitic Asthma |
|  | bronchus asthma |
|  | immediate asthmatic response |

---

|  |  |
| --- | --- |
| MeSH ID | D001249 |
|  | M0001885 |

---

|  |  |
| --- | --- |
| MeSH Heading | Asthma |

---
